# Bridging the digital divide: Understanding COVID-19 diagnostic and vaccination experiences in a socioeconomically disadvantaged neighborhood in Sweden

**DOI:** 10.1186/s12913-025-13033-9

**Published:** 2025-07-01

**Authors:** Rathi Ramji, Dipak Surie, Stefan Cirovic, Margareta Rämgård, Sergey Shleev, Anders Kottorp

**Affiliations:** 1 Department of Care Science, Health and Society, Malmö University, Malmö, 20506 Sweden; 2 Citizen Health Research Center, Malmö University, Malmö, 20506 Sweden; 3 Department of Computer Science and Media Technology, Malmö University, Malmö, 21119 Sweden; 4 Department of Biomedical Science, Health and Society, Malmö University, Malmö, 20506 Sweden

**Keywords:** COVID-19, Commercially available kit, New technology, User perspective, Socioeconomically disadvantaged community, Health promoter

## Abstract

**Background:**

The COVID-19 pandemic had a significant impact on the public health and the economy of the Swedish population, with disproportionate effects on communities living in socioeconomically diverse neighborhoods. To mitigate these impacts and enhance outreach, COVID-19 diagnostic and prevention services supported by digital health tools were introduced for early diagnosis and prevention. Assessing the perceptions related to utilization of these efforts is essential to ensure they are benefiting the particular populations living in socioeconomically disadvantaged neighborhoods. Therefore, the aim of this study was to demonstrate available COVID-19 diagnostic tools and explore the implementation of COVID-19 diagnostics and the digital support services from the experiences of lay health promoters (LHPs) in a socioeconomically disadvantaged neighborhood in Malmö.

**Method:**

Five LHPs participated in an online focus group via Zoom, in May 2021. The session began with an online presentation of testing procedures, followed by discussion to gather user perspectives. The data was analyzed using the Rapid Identification of Themes from Audio recordings method.

**Results:**

Health promoters reported a lack of trust in existing COVID self-test procedures due to validity issues and frequent false-negative results. Polymerase chain reaction testing procedures were deemed inadequate because of delays in receiving results. Additionally, the neighborhood faced barriers to vaccination access, including challenges in using digital technology to book test and vaccination, distance to vaccination centers, and unavailability of slots.

**Conclusion:**

This study highlights the need for affordable and easy-to-use COVID-19 test alternatives in these neighborhoods. The implementation of digital healthcare solutions during the pandemic faced significant challenges, limiting access to care and support in socioeconomically disadvantaged neighborhoods. Therefore, implementing digital healthcare initiatives for disease diagnosis and prevention at the national level requires strategic planning that considers the needs and capabilities of residents in socioeconomically disadvantaged areas. Furthermore, the importance of increasing targeted vaccination centers and educating community representatives, such as health promoters, to better support their communities during crises, was emphasized.

**Supplementary Information:**

The online version contains supplementary material available at 10.1186/s12913-025-13033-9.

## Introduction

Coronavirus disease 2019 (COVID-19) was officially declared as a pandemic by the World Health Organization (WHO) on March 11, 2020 [1]. The COVID-19 pandemic resulted in a serious socioeconomic crisis, economic recession and mass starvation, which particularly affected citizens living in the socioeconomically disadvantaged neighborhood globally and not the least in Sweden [2, 3]. These subgroups frequently experienced compromised health compared to the majority population due to structural issues including poor housing, lack of access to quality education, lack of stable employment opportunities, and barriers to healthcare. These structural issues are often further complicated by social conditions including social exclusion, lack of local support, discrimination, and poverty significantly influencing their health outcomes [4]. These communities are at a higher risk of mental health issues, as well as physical health conditions due to their social circumstances that lead to poor lifestyles [5].

In contrast to other countries in the world including its neighbors, Sweden adopted a distinctive approach in that they did not have enforced restrictions and lockdowns during the COVID-19 pandemic [6, 7]. The Public Health Agency of Sweden (PHAS) prioritized the dissemination of timely, evidence-based information through diverse digital tools and channels, as well as through local authorities and civil society organizations [6, 8]. However, a key limitation of this strategy was the disconnect between national-level decision-makers and communities living in socioeconomically disadvantaged situation [6, 8–10]. Studies from Nordic regions including Sweden have shown that the guidelines and recommendations related to COVID-19 such as health updates, social distancing recommendations and information regarding access to other preventive measures were not adapted to the language and sociocultural barriers faced by the citizens living in disadvantaged neighborhoods [8, 11].

A large proportion of front-line workers were from disadvantaged neighborhoods and often had lower income and limited job security [12]. They relied on public transport as they could not perform their jobs remotely from their homes [12, 13]. Moreover, their unstable employment conditions made it impossible for many to stay confined at home even while experiencing symptoms [8, 12, 13]. Furthermore, the living conditions of these citizens were often compromised owing to structural constraints such as housing shortages and economic challenges forcing them to live in multigenerational households. These conditions posed challenges to follow physical distancing and practice recommended hygiene routines, contributing to the rapid spread of infection [12–14].

On one hand, the already existing challenges in accessing health services appeared to worsen during the pandemic among these communities, as the routine healthcare appointments were almost absent limiting the possibility to receive timely information about prevention and care from their local health care providers [15]. Many citizens in these areas resorted instead to information from their home country or international media. However, those information were not always aligned with the local Swedish context [8, 16]. At the same time, the PHAS primarily used one-way communication to spread information, without considering the unique needs of the citizens [6, 8]. On the other hand, these citizens also lacked trust in the health care system and public health agencies, which created barriers to receiving appropriate information [16]. One of the most significant consequences of this is that despite the situation and the high prevalence of COVID-19, studies show that testing for COVID-19 was relatively lower among those in disadvantaged neighborhoods [17, 18]. Yet, studies exploring the perceptions concerning testing for COVID-19 together with a sudden shift toward digital healthcare services among these populations are relatively sparce, especially in Sweden.

Sweden has an advanced public healthcare system that aims to explore the opportunities offered through digitalization. Digitalization in the Swedish context refers to the integration of digital tools to transform healthcare delivery, enhance efficiency, and empower patients [19]. Digitalization is implemented through the use of digital tools such as national patient portal (1177.se), electronic health records, digital prescriptions, telemedicine and digital consultations within its policy framework “Vision for eHealth 2025” [9, 19].

The core objectives of this framework include:

- Timely, accurate and nation-wide sharing of healthcare information with citizens digitally.
- Protect citizens’ healthcare information while respecting their privacy and ethical concerns through safe and secure information processing and sharing.
- A citizen-centered initiative where the end-users of the digital tools become engaged co-creators who drive the tool development based on their needs, goals, attitudes, limitations, and circumstances.

During the pandemic, the Swedish healthcare authorities implemented digitalization initiatives to conduct COVID-19 diagnosis using digital tools for identification, outcome sharing, and booking vaccinations, in addition to healthcare consultations and social services to improve access and efficiency [15]. Since successful implementation and meaningful adoption of these initiatives relies on both responsiveness to local needs, as well as how the digital support services were offered, it is important to understand the digitalization process. Frameworks such as the Technology Acceptance Model (TAM) [20] that consider the usefulness and ease of use of the digital support services by the citizens are influenced by socio-economic factors and trust in public services. Such a model may offer valuable insights into the conditions that enable or hinder engagement with digital health tools. This may contribute towards understanding if the COVID-19 diagnosis and vaccination services has been inclusive and accessible to the entire population, especially to the citizens living in socioeconomically disadvantaged neighborhoods.

*Thus*,* the aim of this study was to demonstrate the available COVID-19 testing procedures*,* and to explore the implementation of COVID-19 diagnostics and digital support services through the experiences of lay health promoters (LHPs) from a socioeconomically disadvantaged neighborhood in Malmö.*

## Materials and methods

**Fig. 1:**
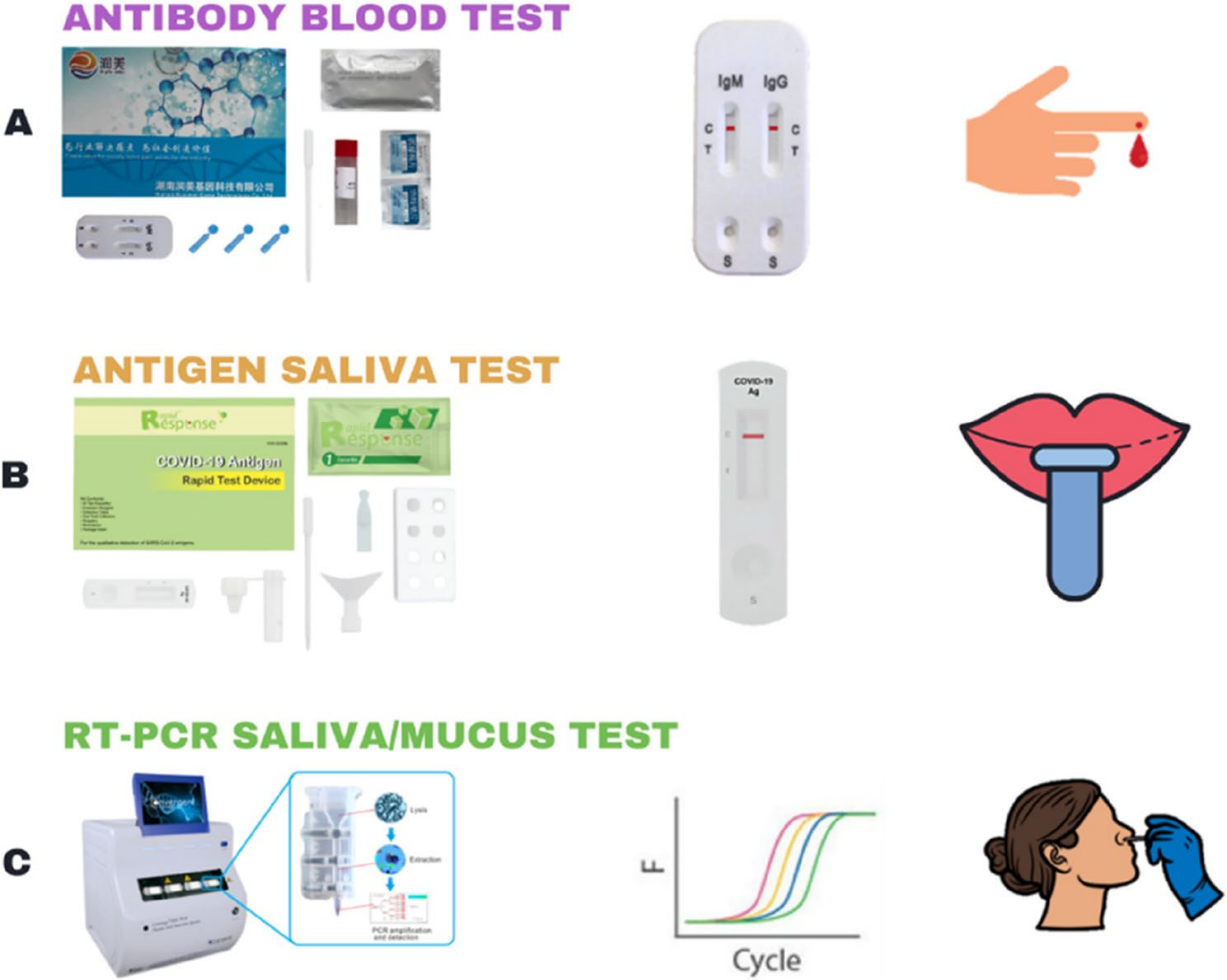
Commercially available kits for COVID-19 diagnostic used in the current study

Commercially available kits for COVID-19 diagnostic, i.e., an invasive blood based nCoV IgG/IgM rapid test, minimally invasive swab based COVID-19 real-time test polymerase chain reaction (RT-PCR) kit, and non-invasive saliva based COVID-19 Antigen Rapid Test (Fig. 1) were acquired from Human Runmei Gene Technology Co., Ltd. (Changsha, China), iNtRON Biotechnology, Inc. (SeongNam-Si, Korea), and BTNX, Inc. (Markham, Canada), respectively.

### Context

This study included residents from a socio economically disadvantaged neighborhood located in the southern part of Malmö, the largest city in the Swedish county of Skåne [21]. The Swedish Reinforcement Agency has classified this neighborhood as one of the fifteen most socially deprived areas in the country, characterized by high rates of unemployment, crime, low educational attainment, and poor health outcomes [22]. Situated approximately 6 km from the city center, the neighborhood is home to around 7,800 residents, and 43% of whom are first- and second-generation migrants. The population includes individuals and families from Iraq, Iran, Syria, Poland, Palestine, Denmark, and the former Yugoslavia [23]. The gender distribution in neighborhood is relatively balanced, with a slight predominance of women, particularly among older age groups. The residents are relatively young with an average age of 30 years, yet an estimated 10–15% of residents are older migrants over 65 years who migrated to Sweden during early 80’s [24]. The average income among employed residents in this neighborhood is less than 70% of Malmö’s average [25]. Despite the presence of essential public services, including primary and dental care facilities, a pharmacy, supermarkets, a library, and social meeting places, nearly 60% of residents report feeling unsafe in public spaces. Moreover, a substantial proportion of the population refrains from utilizing available health and dental care services, citing a lack of adaptation to their linguistic and sociocultural needs [22]. These structural vulnerabilities were further exacerbated during the COVID-19 pandemic. According to a report by the Nordic Council of Ministers, the Fosie district which includes Lindängen was one of the most severely affected urban areas in Sweden with a cumulative COVID-19 incidence of 13.6% in year 2021 [8].

A large program namely “Equal Health - Collaborative Innovations for Health Promotion” [23] based on community-based participatory research (CBPR) was established in 2017 with the goal to promote health and well-being among the residents of the neighborhood. The program initiated by researchers from Malmö University also involved actors from the public, private and non-profit organizations, together with the citizens from the neighborhood. Citizens, together with other stakeholders and academic researchers, were engaged in identifying problems related to their health, as well as planning, implementing, and evaluating health promoting interventions aimed at minimizing health inequalities. The health promotion activities within this program were coordinated by representatives from the neighborhood known as LHPs [26].

### Participants

The five LHPs who were part of the larger health promotion program participated in this study. The LHPs were also residents from the neighborhood and had been part of the program since the beginning when they initially volunteered and were later employed within the program. At a later stage they were employed full-time by some of the non-governmental organisations in the program such as Red Cross, Save the Children and the housing companies participating in the program. All five LHPs were women and came from the countries that represent the citizens living in the neighborhood. Four of the five LHPs were Arabic speaking but also communicated fluently in Swedish. One of the LHP was from Eastern Europe and spoke several Eastern European languages. The LHPs were involved in the planning and facilitation of the different health promoting activities and were also a part of the programs’ steering group as representatives of their community. They were instrumental in inviting and recruiting citizens from neighborhood to participate in the different activities, providing language interpretation, and also communicated the cultural nuances of the community to the research team and the collaborating partners within the program. Above all, the LHPs were bridges between the actors in the program and the citizens given that they were constantly in dialogue with both the citizens and the actors. Apart from the health promotional activities, the LHPs adapted to pandemic regulations and supported the citizens in the neighborhood with practical aspects such as booking COVID-19 test and vaccinations, as well as communicating with health care personnel. They played an important role in bringing forward the community needs that are not usually accounted for, especially during crisis situations.

### Focus group interview

As part of the current study, the LHPs participated in an online focus group discussion facilitated by the research team in May 2021 about 6 months following the introduction of vaccines in Sweden. The discussions were held digitally via Zoom-Video Conferencing owing to the restrictions related to the ongoing pandemic. Zoom has been previously identified as an ideal data collection platform as it is secure, ease to use, and cost-effective [27]. The discussions were preceded by the demonstration of the novel COVID-19 testing procedures combined with a detailed explanation regarding the advantages and disadvantages of these testing methods (Table 1). This was followed by reflective dialogues regarding both the LHPs experiences with the existing COVID-19 testing procedures and supporting digital services as well as their views concerning the tests demonstrated earlier. Two members from the research group moderated the dialogues which lasted for about 50 min, and all discussions were held in Swedish. The discussions were digitally recorded with the consent of the participants. A focus group guide was developed for the purpose of this study and included a series of structured and open-ended questions designed to encourage participants to reflect deeply on their experiences and perspectives. The question guide began with broad questions relating to the situation around COVID-19 in the neighborhood to help participants naturally evolve into the discussion, followed by more detailed questions related to the aim of the study. Reflective questions were also used to prompt deeper insights and personal reflections. Based on LHPs’ responses, additional probing questions were also posed to explore topics in greater depth. The focus group guide is available as a supplementary file.

**Table 1:** Characteristics of commercially available kits for COVID-19 diagnostic

Test	Advantages	Disadvantages
(A) Antibody blood test	• Quick response • Portable	• No direct detection of SARS-CoV-2 • Risk of false negative results • Blood test is required
(B) Antigen saliva test	• Quick response • Portable • Direct detection of SARS-CoV-2 • Non-invasive	• Risk of false negative results
(C) RT-PCR saliva/mucus test	• Non or just minimally invasive • “Gold standard” in COVID-19 diagnostic • Direct detection of SARS-CoV-2	• Long response times • Uncomfortable for some people • Not portable

### Data analysis

The data was extracted from the recorded focus group discussions using rapid identification of themes from audio recordings (RITA) [28], after which a qualitative content analysis with an inductive approach was used to identify relevant themes corresponding to the aim of the study.

The first author (RR) reviewed the recordings repeatedly and developed a content log of the discussions together with a general summary of the discussions. In the next step, codes were rapidly identified through repeatedly listening to the recordings several times. The sixth author (AK), who also led the focus groups listened to the recordings in parallel and repeated the same procedure as the first author. Following this, AK and RR discussed and reflected on their findings together and came to consensus over a final list of codes. The discussion was then confirmed with the rest of the team and the codes were placed under categories. Each of the categories was later defined in detail to identify overarching themes. The themes were subsequently interpreted and discussed through the lens of TAM [20], with particular attention to patterns related to perceived usefulness and perceived ease of use.

### Ethics considerations

The current study was approved by the Swedish Ethical Review Authority (DNR 2020–04063). All activities were performed in accordance with the Declaration of Helsinki. All participants received information about the study. Participation was voluntary and the participants were informed that they could leave the study at any time. Written information about the research process was also provided, and the participants were asked to sign an informed consent form. All materials collected were marked by code, kept confidential, and were accessible only to the research team. Written consent was obtained for publishing the results. They were also informed that the purpose of the research was to investigate their opinion about new and potential COVID-19 testing technology and services, that could potentially be applied in Lindängen.

### Findings

Two main themes emerged while exploring the knowledge, beliefs and perspectives of the LHPs namely ‘Misinformation and mistrust in the diagnostic tools and vaccinations’ and ‘Structural barriers to access diagnostic tools and vaccinations.’ The LHPs believed that the general mistrust and misinformation related to COVID-19 diagnostic tools and vaccinations, coupled with barriers to accessing diagnostic tests and vaccination services, were the primary challenges they faced when supporting citizens in the neighborhood.

### Misinformation and mistrust in the diagnostic tools and vaccination

The LHPs reported that citizens in the neighborhood experienced uncertainty when COVID-19 diagnostic tests and vaccines were introduced without sufficient prior information. This lack of clear communication, combined with fear of stigmatization in the event of a positive test result, led many to refrain from testing. Three subthemes emerged from this overarching theme: uncertainty due to conflicting information, fear of stigmatization, and the need for prior training and information for gatekeepers.

#### Uncertainty due to conflicting information

The LHPs reported that citizens in the neighborhood received different kinds of information from different sources regarding the sensitivity of the COVID-19 diagnostic tests. They were worried that the tests and vaccinations might be fatal. The LHPs believed that the citizens often found information on social media and media channels from their homelands which influenced the choice to test or vaccinate against COVID-19.

*“People have heard from somewhere that it is life-threatening that you can get a fever from vaccination, some think you can die from the vaccination. They have heard from their relatives in their home country” (LHP working with elderly)*

#### Fear of stigmatization

According to the LHPs the citizens in the neighborhood were also afraid to be stigmatized if they tested positive. Some citizens refrained from testing as they could not go to work on being tested positive, which would affect their financial situation. The LHPs also said that some citizens had access to local labs run by acquaintances from their home country that provided false negative test so they could go to work. The LHPs also raised that the availability of false reports further eroded trust in the diagnostic tests among the citizens. The LHPs mentioned that despite being aware of the consequences of symptomatic individuals moving around freely, they felt they lacked the knowledge and support to make these citizens understand the situation.

*“Most people in my job come with fake certificates but they did have symptoms, they had Corona. I know it is life threatening, but they don’t want to be unemployed” (LHP working with children families)*

The LHPs mentioned that the few citizens who initially tested themselves found that the test was not reliable. Some of their family members who exhibited common symptoms of COVID-19 received negative test results. However, when they tested themselves for antibodies a few weeks later, it appeared that they had previously had COVID-19. This made them question which of these tests were actually genuine and thus, refrained from testing themselves.

*“The most important thing is that the test shows true results. My husband was infected, he had lost his taste, his smell and had a high fever. We were 99.9% sure that he had Corona, but we tested twice and the first two times it turned out that he did not have Corona as he got negative results twice. But a few weeks later we tried an antibody test, and he had antibodies.” (LHP working with children and families)*

#### Need for prior training and information for gatekeepers

The LHPs were aware that the vaccination was necessary since most of the activities were conducted in groups, and they needed to protect themselves and the other citizens in the group. However, they were not able to prevent citizens who were not vaccinated from participating in the activities. The LHPs highlighted the need for more education and digital support to citizens, particularly during the time of crisis such as the COVID-19 pandemic. The LHPs also said that their own role was challenging because they had to address misinformation while they themselves had limited knowledge regarding the vaccination and diagnostic tests.

*“I must be able to help if they want to test themselves and I must be able to show how to do it. I feel inadequate if I can’t fix my own test. We health promoters need more knowledge” (LHP working with women)*

#### Structural barriers to access diagnostic tools and vaccinations

The LHPs raised that despite the mistrust and the need for more information, some of the citizens did want to test or vaccinate themselves. However, there were structural barriers as practical and systemic obstacles that limited citizens’ ability to access COVID-19 testing and vaccination. Three subthemes emerged from this overarching theme: lack of digital support, physical constrains to assess diagnostic tools and vaccination, and need for reliable and economical diagnostic tools.

#### Lack of digital support

The LHPs highlighted technology as one of the main barriers to access diagnostic tools and vaccination, as all appointments had to be made using the digital identity verification system known as BankID [29]. The LHPs mentioned that digitalization was particularly problematic for older adults and those with functional disabilities, as many of them did not have a digital identity. The elderly with functional disabilities were dependent on their trustees for digital bookings but some of the trustees themselves were sick and/or not accessible.

*“Problems with technology use has existed even before the pandemic. Many in my group do not have the right to have a BankID due to their psychological problems. The system excluded those who do not have the opportunity to do things themselves. We also have people who cannot read and write in my group. There are even people who were born Swedish but have developmental disabilities and have never had technology access. All of them were completely left out” (LHP working with elderly)*

The LHPs stated that even younger families who had access to digital identities found it challenging to book their tests as they were not familiar with the system. There was very limited technical support via telephone which was also often hindered by language barriers. The LHPs reported that the citizens did not understand the procedures and the information material provided together with the COVID-19 self-test kit and they were perceived as insufficient. The LHPs stated that some of the citizens believed that they received a failed test report since the test was not performed correctly. The LHPs themselves sought help from the pharmacist in the neighborhood, but they too lacked knowledge regarding the tests. The LHPs reported that citizens were not aware of other diagnostic tools available in the pharmacies but also stated that these tests were expensive and were not affordable to the citizens in the neighborhood. The LHPs further stated that the citizens also had similar problems booking vaccination as it was also connected to their digital identities.

*“People need a lot of help to book themselves in for vaccination. When they got the letter, they get my help to call their health center and book them in for vaccination. It’s both technology and the language. Everything was only in Swedish, and it was difficult. They also came up with the new app for COVID, but we have people who can’t really understand and it doesn’t help with more technology. The pharmacy or health care do not help with the tests either” (LHP working with children families)*

#### Physical constrains to assess diagnostic tools and vaccination

The LHPs reported that one of the biggest challenges in testing for COVID-19 in Sweden was that only home-tests were available. These tests had to be booked digitally and collected by an asymptomatic person on behalf of the individual being tested. The LHPs highlighted that many citizens, particularly the elderly, lived by themselves. Thus, voluntary social care workers helped the elderly by collecting and sending the tests to health facilities. However, the elderly also needed assistance with the testing procedure itself. The LHPs mentioned that some social workers initially helped the elderly but became infected in the process, and thus there were fewer social workers available in the neighborhood, leaving out many citizens from the possibility of being tested.

*“We were helped by the social workers. The pharmacy could not deliver tests. We needed to write online so that the social workers could help pick up my medicines and tests when we were in quarantine. Many social workers helped the elderly to test and were infected by it. So, the support became lesser and lesser overtime, and we hardly got any thereafter.” (LHP working with the elderly)*

The LHPs said that the vaccinations were provided in limited centers which were far away from the neighborhood. This restricted the access to vaccinations since many of the citizens could not afford the costs for transportation or did not have adequate physical support to commute to the vaccination centers. The LHPs also raised that the booking system was complicated, and they had challenges to find a time slot for vaccination.

*“The problem has been that 50–60-year-olds can get vaccinated only in 5 different health centers around Scania. This means that to be able to get theirs, sometimes they need to travel 30 minutes. It might make it easier if they could get vaccinated in the nearest health center.” (LHP working with elderly)*

#### Need for reliable and economical diagnostic tools

The LHPs said that the citizens as well as themselves were unaware of the availability of alternative testing options other than the one provided by the healthcare system. The LHPs said that the citizens perceived the antibody kit available in the pharmacy as expensive and unreliable, making it neither accessible nor viable for the citizens. The LHPs further highlighted the need for affordable quick tests which they could use with their groups so they can continue their health promoting group activities without the fear of being infected by each other.

*“Most people only have access to the test that is available for free. Many cannot afford to pay SEK 250 for an antibody test. So, they have no idea about other tests. We cannot answer about other tests because we cannot try other testing methods. The public does not have access to everything. It would be nice if we knew more if we had access to some kind of quick test that we can use during our meetings. We all can feel safe that way.” (LHP working with women)*

## Discussion

The results of the study show that the citizens from a socioeconomically disadvantaged neighborhood lacked trust in the available diagnostic tools and prevention measures offered by the Swedish public healthcare system. In addition, digitalization and physical barriers to accessing services were perceived as a burden, further distancing citizens from this neighborhood in utilizing essential healthcare services during the pandemic. Public healthcare services in Sweden strive to both provide the highest healthcare quality to all its citizens, and at the same time reduce the healthcare costs [30, 31]. The choice of healthcare standardization over personalized services leaves the vulnerable population with special needs excluded due to the one-size-fits-all approaches [16, 31]. By examining local experiences of the LHPs, the study aimed to highlight how digital health initiatives can be better aligned with the everyday realities and preferences of communities. While Sweden aims to provide universal ‘health for all’ [32, 33], this study shows that healthcare services appear to primarily benefit certain segments ignoring the heterogeneity of people and their varied needs.

**Fig. 2:**
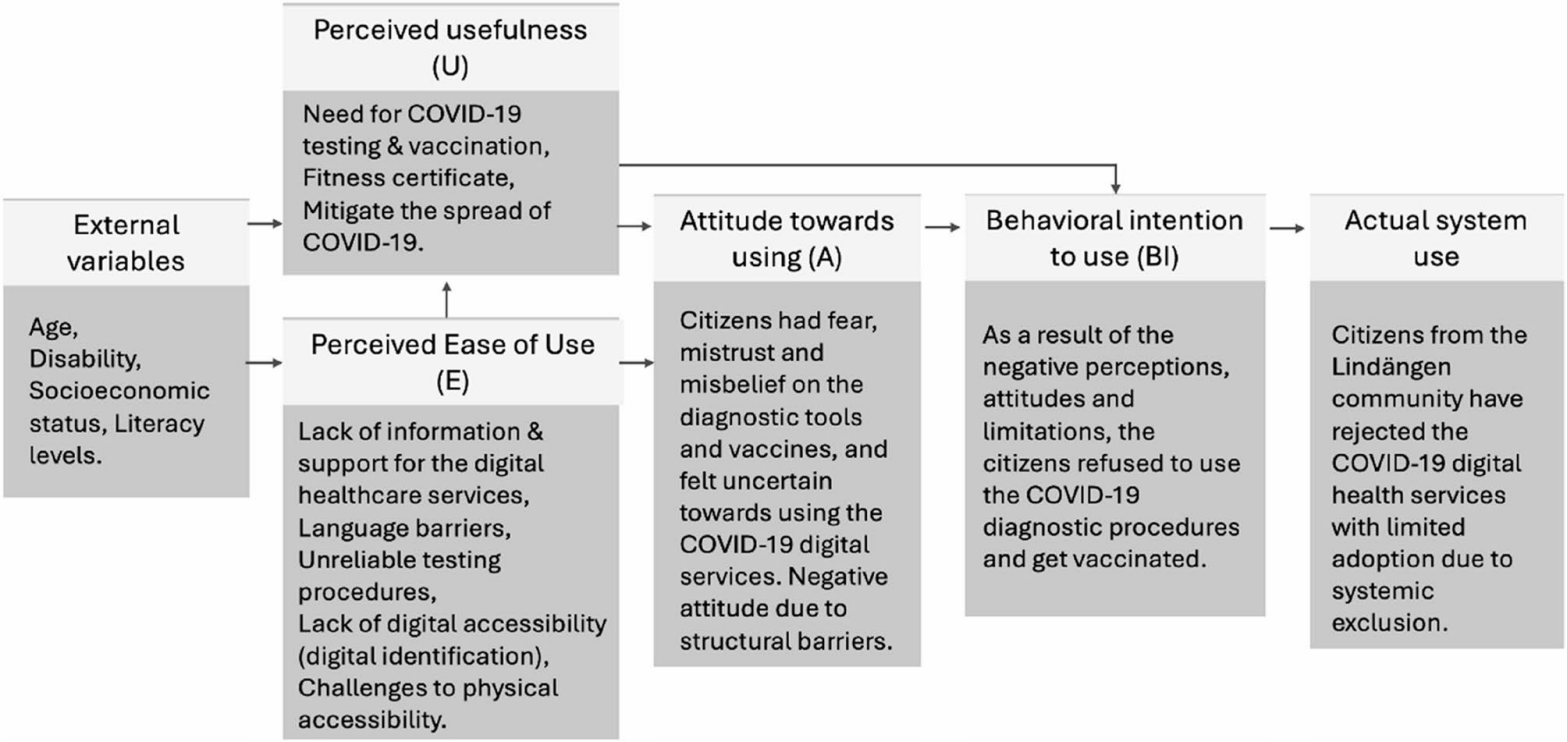
Adapted Technology Acceptance Model (TAM) for COVID-19 digital health services

The study’s findings highlight critical barriers to adopting COVID-19 diagnostic tools and digital health services in a socioeconomically disadvantaged Swedish neighborhood. By applying the TAM framework [20] (refer to Fig. 2), we understood how *perceived usefulness* and *perceived ease of use*, central to technology acceptance were influenced by structural, cultural, and socioeconomic factors. External variables such as age, functional disabilities, socioeconomic status, and the levels of literacy influence the TAM’s ability to explain the adoption and use of COVID-19 digital services. Through discussions with LHPs, it became evident that a large proportion of the population in the neighborhood who needed support during the COVID-19 were elderly, many of whom had functional disabilities limiting them from understanding and accessing the services. This study also showed that language literacy was a key to successful access to the services even among the younger population living in the neighborhood. Findings from a study in Finland among a similar population, reflected identical results, including age-related digital exclusion, language literacy, and socioeconomic barriers as pivotal factors affecting access to digital health services during COVID-19. The Finnish study emphasized that older adults, particularly those with lower education and income levels, faced significant challenges in adopting digital tools, underscoring the need for targeted digital support and inclusive service design [34].

The perceived usefulness of the COVID-19 digital services was undermined by mistrust. According to LHPs, the citizens in the neighborhood questioned the validity of COVID-19 tests (due to false negatives from PCR and rapid test) and the fear around side effects to the vaccines, reducing perceived usefulness. Furthermore, the LHPs stated that misinformation from non-local media exacerbated distrust, aligning with TAM’s emphasis [20] on external variables like cultural context. The lack of tailored communication from Swedish public healthcare authorities as shown in this study seemed to have further eroded trust, a critical factor in determining whether users perceive technology as beneficial. The findings of our study align with previous research from the Nordic regions, which also highlights that the PHAS COVID-19 response in form of information and updates were not adapted to the needs of residents in disadvantaged neighborhoods [8, 11]. Our study reinstates this observation by showing how these barriers significantly impacted individuals’ ability to access and utilize digital health services.

The perceived ease of use of the COVID-19 digital services was hindered by structural barriers. The concerns raised by the LHPs pointing towards digital exclusion due to the reliance on BankID for bookings disproportionately affected elderly, disabled, and linguistically isolated populations, directly impacting ease of use. A previous study on health care workers’ perception on access to healthcare for migrants during COVID-19 also criticized the mandatory requirement of using BankID to book diagnostic tests and vaccinations when population subgroups without access to BankID also existed [15]. While the requirement for fitness certificates underscores the usefulness of the diagnostic services, when reflecting on the perceived ease of use of the digital tools and services offered by healthcare authorities, it becomes evident that there was a lack of understanding of the community citizens’ needs and limitations (e.g. need fake fitness certificates to work and earn money; difficulties in getting the fitness certificates due to mandatory BankID requirement). Furthermore, our study also added that physical barriers identified by the LHPs such as distance to vaccination centers and the lack of transportation (reliance on public transportation) compounded digital challenges, illustration how socioeconomic factors intersect with technology access. A national register-based study on COVID-19 vaccination revealed that vaccine uptake was nearly 20% lower among elderly individuals with lower socio-economic status and predominantly not born in Sweden compared to the general Swedish population [35]. While the national study did not explore the underlying causes of this disparity, our findings suggest that limited physical access to vaccination sites, combined with digital barriers, may have contributed significantly to the lower vaccination rates observed in these populations.

The study revealed the importance of human touchpoints as mediators. Digital tools alone were insufficient and required human support, both in terms of accessing the digital platforms, and to physically assist with collecting the test-kits and submitting the samples for testing. For instance, the LHPs assisted with bookings that were acknowledged as essential for the citizens living in vulnerable conditions. The TAM highlights the need for human mediation in complex socio-technical systems. Similar to findings in the study from Finland [34], Sweden’s digital divide reflects systemic inequities. Vulnerable populations, particularly migrants and low-income groups, face overlapping barriers: lack of e-identification, language gaps, and insufficient trust in institutions. This study uniquely emphasizes the role of local health promoters (LHPs) as cultural mediators.

In this study, negative attitudes toward COVID-19 diagnostic tools and digital services stemmed from perceived uselessness, driven by misinformation and distrust. When tests were perceived as unreliable or vaccines as risky, services seemed to have been rejected by the communities, reflecting a collapse in perceived usefulness. Perceived ease of use was similarly undermined by Sweden’s rigid digital systems because of BankID and other inaccessible digital tools to subgroups of population which signaled systemic exclusion. The gap between intention and usage was worsened by the system’s failure to address intersectional vulnerabilities. These findings underscore that without considering factors like socioeconomic status, age, and literacy, even well-designed technologies risk reinforcing disparities as suggested by previous study on intersectionality [36].

While the European Public Health Association (EUPHA) promotes digitalization to enhance efficiency and empowerment through automation and data analytics [37], this study reveals a disconnect between such goals and the lived realities in marginalized communities. In Malmö’s disadvantaged neighborhood, systemic barriers such as mandatory BankID, language inequities, and distrust in test validity excluded migrants, older adults, and low-income groups. Although the WHO emphasizes inclusive planning [38], Sweden’s approach highlights the need for participatory co-design with, not just for, marginalized groups. The “Equal Health” program [16, 23, 26], grounded in CBPR [39], exemplifies this approach. LHPs acting as cultural and linguistic bridges, embody CBPR’s principle of equitable partnership by facilitating digital access and countering misinformation [16, 26].

Engström et al. [40] advocate for segmentation to design citizen-centric services based on individual and contextual factors. This aligns with our findings: structural inequities like digital exclusion and distrust shaped distinct behaviors such as test avoidance and reliance on intermediaries. These behaviors limit community citizens’ ability to co-create value with health systems. Segmentation also clarifies how sociocultural and socioeconomic factors influence perceived usefulness and usability—core to TAM. For instance, citizens with limited digital literacy (Engström’s “high support, low autonomy” segment) depended on LHPs, revealing a mismatch between uniform digital systems and diverse community needs. Meanwhile, “self-reliant” residents sought informal alternatives, though their agency was often constrained by affordability and language barriers.

### Limitations

This study included a small and homogeneous sample of LHPs which may be seen as a limitation. However, the perspectives of the LHPs are grounded in their extensive experience working with citizens in the neighborhood throughout the pandemic, regardless of age, gender, or other social factors. The results of the study also highlighted participants’ perceptions around vaccination, although this was not part of the original study aim and may be viewed as a limitation. While no specific questions about vaccination were included in the focus group guide, the topic emerged organically during discussions and was explored further through probing. This illustrates how participants experienced COVID-19-related services as interconnected with vaccination services. rather than as isolated elements. Instead of undermining or blurring the study’s focus, this spontaneous inclusion enriched the findings and aligned with the broader implementation context.

Furthermore, the results should, however, be interpreted with caution, particularly regarding the experiences of older adults, as they were not interviewed directly. Nevertheless, the LHPs have been working with citizens in the neighborhood, including the elderly, long before the pandemic. They have established trust-based relationships that continued throughout the pandemic. The LHPs were in close contact with the elderly, providing support and witnessing their struggles and experiences firsthand, and thus could provide authentic accounts on behalf of the elderly. The findings of the study on the usability of digital healthcare services may have been negatively impacted by the ongoing pandemic. However, the results also indicate that the sudden introduction of digital healthcare services during a crisis can pose significant challenges for vulnerable populations.

## Conclusion

This study exposes a core paradox in digital public health: while digitalization aims to democratize care through automation and empowerment, it can deepen inequities if structural barriers remain unaddressed. Based on findings from a disadvantaged Swedish neighborhood during COVID-19, the research shows how digital exclusion (e.g., mandatory BankID), language barriers, and institutional distrust weakened digital health efforts. These issues stemmed from deeper sociocultural and economic inequalities, influencing perceptions of usefulness and ease of use. Applying the TAM, the study frames technology adoption as a sociotechnical process shaped by trust, access, and cultural relevance. Misinformation and limited test reliability reduced confidence, while rigid digital systems excluded those lacking digital access or literacy. LHPs emerged as key intermediaries, bridging institutional systems with local needs—highlighting the value of human touch in experiencing digital services. To ensure equity, policymakers must prioritize participatory co-design, support non-digital alternatives, and build trust through sustained community engagement. These steps are essential—not optional—for ethical and effective digitalization in healthcare.

## Supplementary Information

Supplementary Material 1.

## Data Availability

The data that support the findings of this study are available from the research group at Malmö University, but restrictions apply to the availability of these data, obligated by the Swedish Ethical Review authority’s guidelines and Malmö University’s GDPR protocols and so are not publicly available. The data are, however, available from the authors upon reasonable request and with the permission from the concerned.
