# Supplementary material for: Bridging the digital divide: Understanding COVID-19 diagnostic and vaccination experiences in a socioeconomically disadvantaged neighborhood in Sweden
Source: BMC Health Serv Res. 2025 Jul 1;25:870. doi: 10.1186/s12913-025-13033-9 (PMC12220538; doi:10.1186/s12913-025-13033-9)
Supplement: Supplementary file 1 — Supplementary Material 1. [file 12913_2025_13033_MOESM1_ESM.docx]

**Guide for focus group with Lay Health Promoters**

**Experiences of COVID-19 diagnosis and digital health services during the pandemic**

***Opening questions***

1. What concerns have come up during last year about COVID19 tests in the communities you are in contact with.
2. Have concerns changed over time?

***Reflective questions***

1. What are your own experiences with testing during the pandemic? Where have you done it? Have you tried different types of tests?
2. “Some tests are easy to use as home tests, while others require lab testing”. What are your community members experiences with these? Pros and cons? Which of them are perceived reliable?
3. “Some tests are designed to draw samples without any level of discomfort, while others require drawing blood or deep nasal swabs and thus can be uncomfortable but are at the same time more reliable.” How has these been perceived and used by the community members?

***Detailed questions***

*Access and Usability*

1. How do you and your community currently use the digital health services offered during COVID-19 pandemic? What devices (smartphones, tablets, computers) do you use, and are they reliable with internet access?

*Challenges and Inclusivity*

1. What challenges, if any, do you or others face when using these digital health services, such as language barriers, technical skills, or navigating the tools?

*Needs and Gaps*

1. Are there any additional health-related digital services that you feel are missing or needed to better support your community's self-care and health monitoring during the COVID-19 pandemic?

*Benefits and Barriers*

1. What benefits have you observed from the digital health services, and what limitations or barriers make it difficult to use them effectively?

***Concluding question***

1. What solutions or changes do you think would help your community better use diagnostic and digital health tools during emergency times like the COVID-19 pandemic?
